# Adherence to Mediterranean Diet Among Prediabetic Patients in East Jerusalem

**DOI:** 10.3390/nu17111777

**Published:** 2025-05-23

**Authors:** Aya Zuaiter, Vered Kaufman-Shriqui, Samir Zuaiter, Dima Bitar, Lina Zuaiter, Orly Manor, Ora Paltiel, Amnon Lahad

**Affiliations:** 1 Braun School of Public Health and Community Medicine, Hebrew University of Jerusalem, Jerusalem 91120, Israel; lina.zuaiter@gmail.com (L.Z.); orlyma@ekmd.huji.ac.il (O.M.); opaltiel@gmail.com (O.P.); 2 Department of Nutrition Sciences, Faculty of Health Sciences, Ariel University, Ariel 4076405, Israel; 3 Clalit Health Services Jerusalem District, Jerusalem 97103, Israel; samirzu@clalit.org.il (S.Z.); bitard@clalit.org.il (D.B.)

**Keywords:** body mass index, mediterranean diet, prediabetes, smoking, socioeconomic status, waist circumference

## Abstract

Background: Prediabetes, a precursor state to type 2 diabetes mellitus (T2DM), is characterized by elevated glucose levels that are not yet in the diabetic range. It is often associated with comorbidities such as obesity, hypertension, and dyslipidemia, driven by unhealthy lifestyle factors. This study aims to assess the relationship between adherence to the Mediterranean diet and anthropometric measures, such as body mass index and waist circumference, in Arab adults with prediabetes, considering other lifestyle patterns, including smoking, socioeconomic status, and physical activity. Methods: We performed baseline data analysis among a sample of prediabetic participants of a clinical trial aimed at improving physical activity and healthy lifestyle behaviors. Patients were recruited from the Sheikh Jarrah Clalit Health Services clinic in East Jerusalem. Eligible participants were identified via medical record review and invited by their primary physician. After providing informed consent, participants completed interviewer-administered questionnaires covering sociodemographic data, physical activity, and dietary habits. Physical measurements, including height, weight, and waist circumference, were taken using standardized protocols. Adherence to the Mediterranean diet was assessed using the locally adapted Israeli Mediterranean Diet Adherence Screener (I-MEDAS). Results: A total of 172 prediabetic adults aged 40–69.9 years were recruited. The majority of participants exhibited high adherence to the Mediterranean diet, with 80.2% achieving a high adherence score. However, no significant associations were found between Mediterranean diet adherence and BMI or waist circumference. Active smokers were 70.6% less likely to adhere to the Mediterranean diet compared to nonsmokers, and participants with equal-to-average income had lower odds of adhering to the diet compared to those with below-average income. Conclusions: These findings underscore the need for tailored public health strategies that address local cultural, economic, and environmental factors influencing dietary habits. Improving adherence to the Mediterranean diet in this population will require a multifaceted approach, with further research needed to understand the complex relationship between diet, lifestyle, and chronic disease prevention.

## 1. Introduction

Diabetes mellitus is a chronic metabolic disorder characterized by elevated blood glucose levels, leading to significant health complications if unmanaged. Prediabetes, a precursor state to type 2 diabetes mellitus (T2DM), involves impaired glucose tolerance or fasting glucose levels that are higher than normal but not yet in the diabetic range [1]. The prevalence of prediabetes is rising globally. The International Diabetes Federation (IDF) estimated that approximately 635 million adults aged 20–79 worldwide are prediabetic, representing about 1 in 8 adults [2]. In Israel, prediabetes affects 5.4% of the population, with higher rates among Arabs, particularly Arab women aged 55–64, where 42% show evidence of impaired glucose metabolism—double the rate observed in other populations [3].

Environmental and lifestyle factors such as obesity, physical inactivity, poor diet, and smoking significantly increase the risk of developing prediabetes, while behavioral factors like sedentary lifestyle and unhealthy eating habits further exacerbate the likelihood of developing these conditions. The American Diabetes Association highlights that obesity, particularly abdominal obesity, and a family history of diabetes are critical risk factors for both prediabetes and type 2 diabetes [4,5]. Nutritional transition and the adaptation to a Western diet have been associated with an increased risk of developing diabetes and prediabetes [6]. Evidence has revealed that Mediterranean countries have evolved mainly from a plant-based diet to an animal-based one, indicating a westernized food pattern and a move away from a traditional Mediterranean diet [7,8]. The Western diet is defined by high intake of fats (particularly saturated fat and hydrogenated and polyunsaturated vegetable oils), high intakes of sugar and sugar-sweetened beverages (SSBs), refined grains, animal-based food, red and processed meat, but low consumption of wine, cruciferous vegetables, and yellow vegetables. The transition from traditional to modern lifestyles over the past century has also been accompanied by decreased daily physical activity, which is, in itself, a risk factor for the development of obesity and prediabetes [7].

The Mediterranean Diet (MedDiet) was first described from observations on people’s dietary habits in different regions of the Mediterranean basin [9,10]. The MedDiet is generally characterized by a high intake of plant foods (e.g., vegetables, fruits, legumes, nuts, preferably whole-grain cereals), use of olive oil as the main dietary fat, a moderate intake of dairy products, a low or moderate intake of meat and fish, and a moderate consumption of wine with meals [11]. Although fruits, vegetables, cereals, and olive oil are common elements in most definitions of the MedDiet, the core diet has many local variations/adaptations across the Mediterranean region. Each country of the Mediterranean basin has its culinary customs influenced by specific socio-cultural, religious, and economic factors. Thus, fermented beverages in the MedDiet, such as wine and beer, are excluded from the dietary pattern in Muslim countries, but there is a higher consumption of tea and fruit juices [11,12]. Scientific evidence supports the positive health benefits of the MedDiet in minimizing the risk of all-cause and cause-specific mortality [13]. It has been recognized for its association with the reduced risk of developing several chronic and degenerative diseases, including various types of cancer, type 2 diabetes, and cardiovascular diseases [12,13].

An Israeli study that examined adherence to the MedDiet using a modified KIDMED index in adolescents based on a nationwide health and nutrition survey (Mabat Youth Survey), 25.5% of the total participants had poor adherence to the MedDiet, 55.2% had average adherence, whereas 19.3% had good adherence to the MedDiet [14]. Another study estimated the nutrient intake of three different Mediterranean populations (Spain, Morocco, and Palestine) and their adherence to the MedDiet; the Spanish participants showed closer compliance to the MedDiet recommendations than the diet of the Moroccans and Palestinians. In reference to the Palestinian population, adherence was 6.36 fold higher in the Spanish population and 3.88 fold higher in the Moroccan population [15]. Scientific studies on adherence to the Mediterranean diet among prediabetic individuals in the Arab population are limited. However, one study conducted in the Middle East and North Africa (MENA) region investigated the role of the Mediterranean diet in managing type 2 diabetes. Findings suggest a declining adherence to the Mediterranean diet in these populations, accompanied by a shift toward diets high in processed foods and refined carbohydrates, which may increase the risk of developing type 2 diabetes [16].

The main objective of this paper is to examine the association between adherence to the Mediterranean diet and anthropometric measures such as BMI and waist circumference among Arab adults with prediabetes. Higher adherence to the Mediterranean diet is hypothesized to correlate with lower BMI and waist circumference. The null hypothesis suggests no associations between diet adherence and these body measurements.

The secondary objectives of this study include examining the relationship between adherence to the Mediterranean diet and various demographic and lifestyle factors. Specifically, the study aims to assess adherence by gender, education level, occupation, income level, smoking status, physical activity, and age.

## 2. Materials and Methods

### 2.1. Study Design

This is a baseline data analysis based on the data collected from participants in an intervention study titled “Improving Physical Activity Levels in the Arabic-Speaking Residents of East Jerusalem with Prediabetes” (NCT03821220).

### 2.2. Inclusion and Exclusion Criteria

The eligibility criteria required participants to be between 40 and 69.9 years old and capable of communicating and understanding Arabic. Eligible individuals also had to have a fasting blood glucose level between 100–125 mg/dL or an HbA1C between 5.7–6.4% at least twice in the past two years, indicating prediabetes. Additionally, participants had to provide written informed consent and be members of Clalit Health Management Organization, attending the Sheikh Jarah clinic.

Exclusion criteria included a diagnosis of diabetes, as evidenced by recorded medical diagnoses or recent fasting blood glucose values above 126 mg/dL or HbA1C over 6.5%. Diabetes treatments beyond metformin were excluded. Since the trial intervention focused on physical activity, other exclusions were made for participants with artificial lower limbs, those unable to walk normally (such as due to a stroke with plegia), chronic obstructive pulmonary disease (COPD), or a history of heart disease. Exclusion criteria also encompassed pregnant individuals, those who had recently experienced a cardiac event, transient ischemic attack (TIA), or cerebrovascular accident (CVA) with residual impairments, as well as individuals who had undergone surgery necessitating hospitalization within the previous year. Patients with active malignancies, except cases of non-melanoma skin cancer, were likewise excluded.

### 2.3. Ethical Aspects

The study received ethics approval number 0212-17-COM2 of the Helsinki (IRB) committee from Clalit Health Services in collaboration with the Hebrew University of Jerusalem. NCT study number: NCT03821220.

### 2.4. Study Procedure

The clinic’s medical records were reviewed by the Sheikh Jarrah Alef Clalit Health Service clinic manager to identify potentially eligible participants based on age and impaired fasting glucose results from the past two years. These patients were then invited to the clinic for a consultation with a primary physician, who explained the study. Informed consent was obtained from all participants.

### 2.5. Assessment

At baseline, each participant was interviewed to complete the questionnaires, including sociodemographic, anthropometric, and dietary surveys. All data were reported through the in-person interviewer-administered survey. Operational definitions for different variables are shown in Appendix A, Table A1.

The original study questionnaires in Hebrew were translated into Arabic and back-translated by a certified translator. In-person interviews were conducted at the clinic and in the participant’s native language by trained interviewers. Weight and height were measured twice, and the average of the two measures was recorded.

Height was measured to the nearest centimeter using a calibrated measuring rod while the participant stood barefoot. Weight was assessed with a portable electronic scale from the healthcare brand Shekel, with participants wearing minimal clothing and barefoot. Body Mass Index (BMI) was computed as weight in kilograms divided by the square of height in meters (kg/m^2^). Waist circumference (WC) was measured using a non-stretch tape measure to the midpoint between the lowest rib and the top of the hip bone. Weight, height, and waist circumference were measured twice to calculate the average for more accurate results.

#### Dietary Assessment

**Figure A1:**
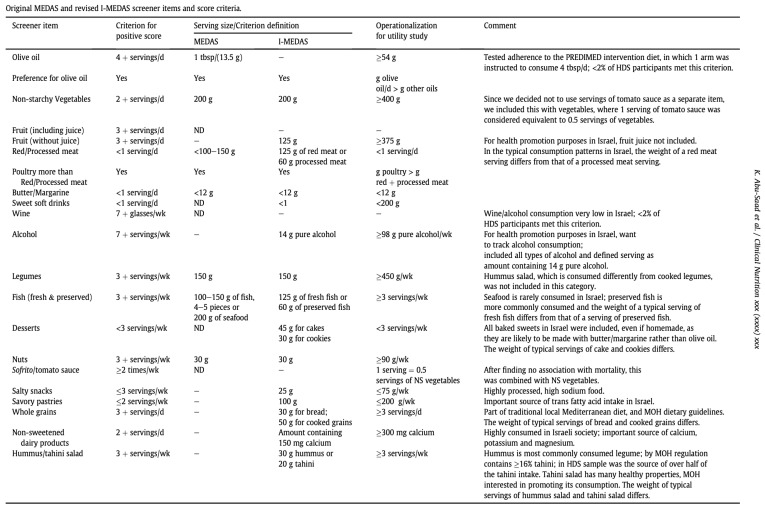
Adaptation of original MEDAS. Adapted from Abu-Saad et al. (2019) [17].

The Israeli Mediterranean Diet Adherence Screener (I-MEDAS) [17] was used to assess adherence to the local version of the Mediterranean diet. I-MEDAS is an adaptation of a 14-item MEDAS, which is designed for use in the Prevención con Dieta Mediterránea (PREDIMED) trial in the Spanish population to assess the long-term effects of the Mediterranean diet on incidents of cardiovascular disease (CVD) [17]. Local adaptations were made to MEDAS based on national dietary recommendations and dietary intake data for the Israeli population. Appendix A, Figure A1 presents the original 14-item MEDAS scoring components and a summary and rationale for the adaptations made to the items for I-MEDAS. After the adaptations, I-MEDAS included 17 components. Servings were translated into grams using the same definitions as used in the I-MEDAS score when available and appropriate to calculate the score. For the current study, researchers decided not to use the question regarding alcohol consumption, as the research was in a Muslim population. Each component received a score of 1 if the criterion was met and zero if not; therefore, the total I-MEDAS score ranged from 0 to 16 points.

### 2.6. Statistical Analysis

The sample size calculation was designed to determine whether the correlation coefficient between the Mediterranean diet score and BMI differs from zero. We applied a moderate effect size of r = 0.3, two-sided of α = 0.05, and 80% power, resulting in a minimum sample size target of 85 participants. If the study power had increased to 90%, we would have needed a minimum sample size of 113 participants.

Data analysis was conducted using IBM SPSS V.29. Descriptive statistics were performed for all variables, with categorical variables described in frequency (*n*) and proportions (%), using Pearson’s Chi-Square test. Continuous variables were summarized by their mean and standard deviation. The normality of constant variable distributions was assessed using the Kolmogorov-Smirnov test.

The baseline scores of I-MEDAS, expected to have a non-normal distribution, were described using nonparametric statistics, specifically the median and interquartile range (IQR). Each component of the Mediterranean diet was categorized into binary categorical variables according to criteria for a positive score (as detailed in Appendix A, Figure A1). Associations between the level of adherence to the Mediterranean diet (as a categorical variable) and continuous variables were evaluated using the non-parametric Mann-Whitney U test. Pearson’s chi-squared test assessed associations between diet adherence and other categorical variables.

Finally, a binary logistic regression model was used to assess the association between adherence to the Mediterranean diet and BMI, waist circumference, income level, and smoking status. This model controlled for potential confounders, including age and sex.

## 3. Results

Table 1 presents the sociodemographic and weight-related characteristics of 172 study participants categorized by the Israeli Mediterranean Diet Adherence Screener (I-MEDAS) score. The sample included a higher proportion of women (67.4%), with a mean age of 55 ± 8. The majority (52.9%) were unemployed. Most participants (56.4%) had an income below the national average. Among those with low adherence to the Mediterranean diet, 47.1% reported below-average income, while 10.3% reported above-average income. Twenty-nine percent of participants were current smokers. Among them, 58% were men and 42% were women. Among participants with high adherence to a Mediterranean diet, 64.4% were nonsmokers, 21.2% were current smokers, and 11.5% were previous smokers. When comparing participants among smoking status, 42.2% of the participants who reported active smoking had lower adherence to the Mediterranean diet. A higher percentage of participants with high adherence to the Mediterranean diet were nonsmokers (64.4% vs. 45.6% among participants with low adherence to the Mediterranean diet, *p* < 0.05). Most participants (73%) answered “no” when asked whether they engaged in physical activity at least once a week. When measured in terms of steps, the median number of steps for the study sample was 5882 steps per day (IQR = 3661).

Six point four percent of the study participants had a normal body weight (BMI between 18.9 and 24.9 kg/m^2^), while 31.4% were classified as overweight (BMI between 25.0 and 29.9 kg/m^2^), and 62.2% were considered obese (BMI greater than 30 kg/m^2^). None were underweight. There was no significant correlation between adherence to the Mediterranean diet and body mass index (*p* > 0.05).

Regarding waist circumference, 25.7% of participants had measurements below the cutoff (females < 88 cm, males < 102 cm). In comparison, 74.3% had measurements within or above the cutoff (88 cm or more for females, 102 cm or more for males).

Table 2 provides detailed information on the dietary characteristics of the study population, focusing on adherence to various components of the Mediterranean diet. The median score of the total sample was 9, which corresponded to a high adherence to the Mediterranean diet (MedDiet). 80.2% of the individuals had high adherence to the MedDiet, followed by 19.2% with medium adherence, with the lowest percentage corresponding to low adherence to the MedDiet (0.6%). The vast majority (94.8%) of participants used olive oil as their primary source of oil. Over half (56.3%) of participants consumed more poultry/white meat than red meat. Overall, the results suggested that this population generally adhered to a Mediterranean-style diet, with high use of olive oil, adequate intakes of fruits, vegetables, and unsweetened dairy, and relatively low intakes of red/processed meat, sweetened beverages, and baked goods. However, there was room for improvement in increasing whole grain, legume, fish, and nut consumption.

Table 3 provides information on adherence to various components of the Mediterranean diet, comparing individuals based on their BMI (obese vs. non-obese, BMI ≥ 30 kg/m^2^ vs. BMI < 30 kg/m^2^) and waist circumference (below vs. at or above the cut-off, 88 cm or more for females, 102 cm or more for males). The percentage of participants consuming nuts (equal to or more than three servings a week) was higher among non-obese participants and participants with normal waist circumference (63.1% and 59.1%, respectively). However, these differences among groups were marginally significant (*p* = 0.065). The table indicated that participants consumed a high amount of olive oil, vegetables, and white meat and poultry. However, only a small percentage consumed fish (7%), legumes (9.3%), and whole grain products (18.2%) at the recommended levels.

Table 4 presents the binary logistic regression model of adherence to the Mediterranean level and possible independent factors influencing it. The model explained 13% (Nagelkerke R^2^) of the variance in adherence to the Mediterranean diet and correctly classified 60.2% of cases. The following variables were entered into the regression model: sex, age, waist circumference, BMI, income level, and smoking status. Despite the lack of a statistically significant association between income level and the outcome as a univariate variable, this variable was kept in the model. The decision to retain it was based on its *p*-value of 0.07, which fell just short of the conventional threshold for significance. This borderline *p*-value suggested that income level may still have had some meaningful influence on the outcome, even if it didn’t meet the strict criteria for statistical significance. Moreover, although age and sex were not significantly associated with the outcome, they were retained in the model as universal covariates. Body mass index and waist circumference were kept in the analysis, even though they did not show a statistically significant effect on the outcome, as they were used to test the study hypothesis. Compared to individuals with income equal to the average, those with below-average income were significantly 2.7 times more likely to adhere to the Mediterranean diet (OR = 0.37, 95% CI: 0.18–0.77, *p* < 0.05). Conversely, those with above-average income were 21% less likely to adhere to the Mediterranean diet than those with below-average income, though this result was not statistically significant.

Individual component scores also examined adherence to the Mediterranean diet, comparing those with below-average income to those with average income. We found that participants with average income were more likely to consume less than seven servings of red or processed meat per week (8.9%) and three or more servings of fish per week (10.7%) compared to those with below-average income (2.1% and 3.1%, respectively; *p* = 0.05 for both). Additionally, those with average income were more likely to consume less than three servings of salty snacks per week (16.1%) compared to those with below-average income (6.2%, *p* = 0.048) (Supplementary Table S1).

The model indicated that income level and smoking status significantly impacted adherence to the Mediterranean diet (*p* < 0.05 and *p* < 0.004, respectively). Smokers were less likely to adhere to the Mediterranean diet than nonsmokers. Active smokers had 70.6% lower odds of adherence to the Mediterranean diet than nonsmokers (95% CI: 0.130–0.663). In summary, smoking status and income level were the most important predictors in this model. The specific categories of these variables, active smokers and income equal to the average, showed significant negative associations with adherence to the Mediterranean diet. Other variables, including age, sex, waist circumference, and BMI, did not show statistically significant associations in this model.

## 4. Discussion

The study assessed baseline adherence to the Mediterranean diet among Muslim-Arab adults with prediabetes who participated in a randomized clinical trial to increase physical activity among individuals from East Jerusalem. Overall, the median Mediterranean score was 9, indicating moderate to high adherence to the Mediterranean diet. In contrast, a 2021 systematic review by Obeid et al. found that most populations in Mediterranean countries have shown low to moderate adherence to the diet over the past decade [18].

The primary objective of the study was to examine potential associations between adherence to the Mediterranean diet, BMI, and waist circumference. Findings on the association between the Mediterranean diet and weight status are mixed. While a 20-year prospective study of 1582 Greek adults reported a significant inverse relationship between adherence to the Mediterranean diet and both BMI and waist circumference [19], and a recent review and meta-analysis of cohort studies found that the Mediterranean diet is associated with a significantly lower risk for elevated BMI, however it showed moderate heterogeneity across findings [20].

In contrast, a large cohort of 27,544 Nordic women, aged 29–49, and an additional cohort of 4162 post-menopausal women from the UK, showed no association between BMI and adherence to the Mediterranean diet, despite positive impacts on abdominal obesity [21]. In studies with an inverse association between Mediterranean diet and BMI, in extensive cohort studies, specifically the EPIC studies, it is worth noticing that the effect was major among healthy, younger individuals with normal weight [22,23].

In the present study, involving prediabetic adults aged 55 ± 8 years, no significant associations were observed between Mediterranean diet adherence and BMI. This finding aligns with some studies involving older or metabolically compromised populations, where the expected impact of dietary adherence on BMI is less pronounced. Several factors may explain the lack of significant associations in this study. The patients with diabetes included were those whose family physicians had already informed them about their health condition and the potential consequences of not improving their lifestyle. While participants may have recently begun adopting a Mediterranean diet, the impact on BMI and waist circumference and its impact on BMI and waist circumference may not yet be observable. Additionally, because the dietary interviews were conducted individually with researchers, many of whom were dietitians, social desirability bias is possible. Participants may have adjusted their responses when speaking with nutrition professionals. The study might not have captured long-term dietary habits, which are more likely to influence BMI and waist circumference. Additionally, the relatively small sample size of 172 participants, coupled with the randomized clinical trial design, may have limited the study’s power to detect significant associations between Mediterranean diet adherence and BMI.

The study’s secondary objective was to examine other predictors and covariates associated with adherence to a Mediterranean diet, including age, sex, education level, occupation, income level, smoking status, and exercise. Among these, only smoking status and income level showed statistically significant associations with adherence to the Mediterranean diet. Our results align with past studies conducted in Greek and Spanish populations. Current smokers were less likely to adhere to the Mediterranean diet than nonsmokers [24]. More generally, the association between smoking and an overall poorer diet has been observed in other populations as well.

The observed associations between income and adherence to the Mediterranean diet in our study contrast with previous research that typically associates higher income levels with greater dietary adherence. For instance, Bonnacio M. et al. found a significant positive association between higher household income and greater adherence to the Mediterranean diet (*p* < 0.0001), with higher income levels correlating with increased adherence [19]. Similarly, a meta-analysis by Kontogianni et al. reported that higher income was consistently associated with improved adherence to the Mediterranean diet, driven by greater affordability of nutrient-dense foods like nuts, fish, and olive oil [25]. In contrast, our study suggests that individuals with lower than average income were more likely to adhere to the Mediterranean diet than those at the average income level. This may be related to variability in the affordability and availability of Mediterranean diet components within this population. This discrepancy may reflect contextual factors unique to our study population, where economic constraints may limit access to more expensive, processed foods while promoting the consumption of traditional, home-cooked meals that align with Mediterranean diet principles. Lower-income populations may also prioritize less costly staples such as legumes, grains, and vegetables, which are core components of the Mediterranean diet but are less frequently consumed by higher-income groups opting for convenience foods.

Furthermore, the finding that participants with average income reported higher consumption of salty snacks and lower intake of legumes may suggest that those with moderate economic resources are more likely to purchase processed foods, potentially due to time constraints associated with employment or perceived convenience. This pattern aligns with findings from the PREDIMED trial, which noted that moderate-income individuals tended to consume more packaged and convenience foods, deviating from the traditional Mediterranean diet [26].

As detailed in Table 2, participants generally adhered to a Mediterranean-style diet characterized by high use of olive oil, adequate intake of fruits, vegetables, hummus, and tahini, and relatively low consumption of red/processed meats, sweetened beverages, and baked goods. However, there is room for improvement in increasing the consumption of whole grains, legumes, fish, and nuts.

Traditional Arab foods such as Hummus and falafel, commonly consumed as breakfast items, are widely available and affordable, contributing to many families’ frequent consumption of foods in East Jerusalem. Likewise, the widespread availability and relatively low cost of fresh fruits and vegetables facilitate their regular consumption. Additionally, the cultural tradition of participating in the annual olive harvest supports the routine use of olive oil. Even individuals not directly involved in harvesting often obtain olive oil locally or from the West Bank at a reasonable price.

Poultry is more affordable than red meat, making it a default choice for many families regardless of economic status. The availability of these budget-friendly options supports adherence to a Mediterranean diet. However, fish, legumes, and whole grains are less affordable and less popular, especially among the older population. Whole wheat bread is not commonly found in traditional bakeries and is often avoided due to misconceptions about its quality. White bread and flour are more affordable, and fish is less accessible due to higher prices and limited availability. Additionally, acquiring fresh fish often requires traveling to other areas, making it less convenient and frequently done by most people.

Another possible reason for our findings is the nutrition transition from a Mediterranean to a Westernized diet. The Western diet is characterized by increased consumption of refined sugars, animal fats, salt, red meats, decreased dietary fiber intake, and fruits, vegetables, whole grains, and unsaturated fats. This dietary pattern contributes to the prevalence of chronic diseases in developed nations [27].

Burgers, fried foods, and pastries are evident among wealthier residents. These dietary shifts are compounded by increasingly sedentary lifestyles supported by car ownership and food delivery services, both hallmarks of a “luxury” lifestyle that may contribute to weight gain. Fast food restaurants are more expensive than staple foods in the area and are less favored by lower-income residents. Consequently, the Western lifestyle tends to be embraced more by those with higher incomes. Additionally, the convenience of a “luxury” lifestyle, including car ownership and restaurant delivery services, promotes a more sedentary way of life, contributing to weight gain.

Interestingly, higher-income individuals showed no statistically significant difference in adherence compared to those with below-average income. This might reflect greater heterogeneity within this group, with some highly adherent individuals but others relying on convenience or Westernized dietary patterns.

Additionally, contrary to what other studies have predicted, our study did not find a significant association between income level, education, employment, and adherence to the Mediterranean diet. In East Jerusalem’s Arab society, having more money does not necessarily correlate with higher education, particularly among older adults. Many people, especially men, do not pursue higher education and instead work in informal, temporary, or part-time work that is not classified as “formal employment” but still generates income, such as working in construction or other high-income jobs that do not require formal qualifications. According to the Palestinian Bureau of Statistics, 14.5% of Arabs aged 15 and older in the Jerusalem governorate hold a bachelor’s degree or higher, 11.6% are unemployed, and 30% are in the labor force [28]. In our sample, 24.4% had a bachelor’s degree, 53% were unemployed, and 24.4% were employed. Our sample had an older age profile and a small size. Moreover, income was measured at the household level rather than individually, so unemployed individuals might still have had access to financial resources from employed family members, especially knowing that most of our sample were women who were considered housewives. In addition, in the Israeli system, most unemployed and retired individuals receive unemployment and retirement benefits or social assistance, which can help them sustain their income temporarily.

Although the study did not assess personal spending, the Palestinian Bureau of Statistics reports that a substantial portion of household income in the Palestinian territories is allocated to food and tobacco, with tobacco expenditures exceeding those for education, healthcare, and personal care. Data shows that the average monthly spending for a household of five in the Palestinian territories in 2017 was 4851 NIS (1348 US Dollars) [28]. Of this, 31% was spent on food (1761 NIS in the West Bank and 1044 NIS in Gaza). The survey also showed that monthly expenditure on tobacco and cigarettes accounted for 5.8% of the total, surpassing spending on education (4.0%), medical care (3.4%), and personal care (1.9%) [28]. This indicates potential public health concerns, as spending priorities may reflect behavioral patterns (e.g., high tobacco use) that could negatively impact health outcomes, and limited investment in health and education.

One major limitation of this study is the small sample size, which may have affected the reliability and robustness of the findings. Recruitment began shortly before the onset of the COVID-19 pandemic, which caused significant disruptions to the study timeline. As a result, participant enrollment and follow-up procedures were suspended, preventing the achievement of the intended sample size. This shortfall may have reduced the study’s statistical power, increasing the risk of Type II errors and potentially obscuring true associations. Consequently, the strength of the observed results may be weakened, and the ability to conduct meaningful subgroup analyses is limited.

## 5. Conclusions

While this study did not find a significant association between adherence to the Mediterranean diet and anthropometric measures such as BMI and waist circumference among prediabetic adults in East Jerusalem, adherence to the Mediterranean diet remains relevant for overall health. Despite the lack of significant weight-related outcomes, the Mediterranean diet is recognized for its beneficial effects on metabolic health, inflammation reduction, and cardiovascular risk mitigation, particularly pertinent for individuals with prediabetes. The findings suggest that a singular focus on body composition may overlook other critical health outcomes influenced by dietary patterns. Consequently, public health strategies should emphasize comprehensive lifestyle interventions that combine dietary guidance with increased physical activity, behavioral support, and long-term adherence to healthy habits. Our findings underscore the need for targeted nutritional interventions considering the socio-economic context of dietary habits. Programs designed to promote the Mediterranean diet in lower-income populations should leverage existing dietary patterns while ensuring access to affordable, nutrient-dense foods. Simultaneously, for average-income populations, interventions may need to address the influence of processed and convenience foods that detract from traditional dietary patterns, emphasizing quick, easy-to-prepare Mediterranean-inspired meals. Further research should explore the intersection of income, food security, and dietary adherence in diverse populations to elucidate the socio-economic dynamics affecting Mediterranean diet adherence.

## Figures and Tables

**Table 1:** Sociodemographic and weight and health-related characteristics of the total sample stratified across adherence to the Mediterranean diet, categorized by I-MEDAS score cut-off.

		I-MEDAS Cut Off						
		Subtotal		Low Adherence to MedDiet (Score Below Median)		High Adherence to MedDiet (Score Equal to or Above Median)		
		Count	Column N %	Count	Column N %	Count	Column N %	*p*-Value
Sex	Males	56	32.6%	24	35.3%	32	30.8%	NS
	Female	116	67.4%	44	64.7%	72	69.2%	
Age, years mean (SD)		55 (8)		54 (7)		55 (8)		NS
Education level	No degree	91	52.9%	40	58.8%	51	49.0%	NS
	High school degree	39	22.7%	14	20.6%	25	24.0%	
	College degree	42	24.4%	14	20.6%	28	26.9%	
Occupation	Unemployed	91	52.9%	40	58.8%	51	49.0%	NS
	Employed	42	24.4%	14	20.6%	28	26.9%	
	Retired	39	22.7%	14	20.6%	25	24.0%	
Income level	Below average	97	56.4%	32	47.1%	65	62.5%	0.07
	Equal to average	56	32.6%	29	42.6%	27	26.0%	
	above average	19	11.0%	7	10.3%	12	11.5%	
Smoking status	Nonsmoker	98	57.0%	31	45.6%	67	64.4%	0.02
	Current smoker	50	29.0%	28	41.2%	22	21.2%	
	Previous smoker	24	14.0%	9	13.2%	15	14.4%	
Exercise once a week (self-reported)	No	126	73.3%	51	75.0%	75	72.1%	NS
	Yes	46	26.7%	17	25.0%	29	27.9%	
Average steps daily	Median (IQR)	5882 (3661)		6082 (3192)		5560.5 (3803)		NS
BMI	Normal weight	11	6.4%	4	5.9%	7	6.4%	NS
	Overweight	54	31.4%	21	30.9%	54	31.4%	
	Obese	107	62.2%	43	63.2%	64	61.5%	
Waist circumference (men and women)	Below the cut-off	44	25.7%	18	26.5%	26	25.2%	NS
	Equal or above the cut-off	127	74.3%	50	73.5%	77	74.8%	

**Table 2:** Dietary characteristics of the study sample (a component of MedDiet *).

Characteristic	Population Value Score (Median (Interquartile Range))
	9.0 (2.0)
MedDiet Component Questions	
Uses olive oil as primary source of oil *n* (%)	165 (94.8)
Eats poultry/white meat more than red meat *n* (%)	98 (56.3)
Vegetable servings/day (median (interquartile range))	2 (1.9)
Fruit servings/day (median (interquartile range))	2 (2)
Butter/margarine/cream servings/day (median (interquartile range))	0 (0.1)
Sweetened beverages/day (median (interquartile range))	0.3 (1)
Whole grain servings/day (median (interquartile range))	0.3 (2.0)
Unsweetened dairy servings/day (median (interquartile range))	2 (2)
Red/processed meat servings/week (median (interquartile range))	2 (2.9)
Legume servings/week (median (interquartile range))	1 (0.75)
Fish servings/week (median (interquartile range))	1 (0.75)
Nut servings/week (median (interquartile range))	3 (6)
Hummus/tahini servings/week (median (interquartile range))	2 (3)
Sweet baked goods servings/week (median (interquartile range))	2 (6)
Savory baked pastries servings/week (median (interquartile range))	1 (1.8)
Salty snacks servings/week (median (interquartile range))	0.1 (1)

* The alcohol item question was omitted from the original I-MEDAS because the Muslim population does not report alcohol consumption.

**Table 3:** Adherence to the components of the Mediterranean diet stratified by BMI and waist circumference cut-off.

	Total (%)	BMI		Waist Circumference			
		Obese (%)	Nonobese (%)	<Cut Off for Both Genders (%)	Equal or Above Cut Off (%)	*p*-Value BMI	*p*-Value Waist Circumference
Use of olive oil as primary source of culinary oil	96	96	100	98	95	NS	NS
Prefer white meat and poultry over red and processed meat	57	60	52	52	59	NS	NS
Vegetables ≥ 2 servings per day	56	58	54	57	57	NS	NS
Fruits ≥ 3 servings per day	34	34	34	30	35	NS	NS
Butter or margarine < 1 serving each day	91	91	92	4	10	NS	NS
Carbonated/sweetened drinks < 1 serving of	60	62	58	36	41	NS	NS
Whole grains ≥ 3 servings per day	19	18	20	16	19	NS	NS
Unsweetened dairy products ≥ 2 servings per day	55	56	54	54	55	NS	NS
Red or processed meat < 7 servings per week	95	96	94	7	4	NS	NS
Legumes ≥ 3 servings per week	9	10	8	11	9	NS	NS
Fish greater ≥ to 3 servings per week	7	6	9	9	6	NS	NS
Nuts ≥ 3 servings per week	54	38	63	59	52	0.065	NS
Humus or tahini ≥ to 3 servings per week	42	38	49	48	41	NS	NS
Sweet baked goods < 3 servings per week	54	51	57	46	46	NS	NS
Savory baked pastries < to 2 per week	77	74	72	20	24	NS	NS
Consume salty snacks < less than 3 per week	91	89	92	89	86	NS	NS

**Table 4:** Binary logistic regression model of adherence to the Mediterranean diet.

Variables	Odds Ratio	95% Confidence Interval	*p*-Value
Sex			
Male (indicator)			
Female	0.89	0.38–2.14	NS
age	1.01	0.97–1.06	NS
Waist circumference			
Below the cut-off (indicator)			
Equal to or above the cut-off	1.48	0.53–4.12	NS
Income level			
Below average (reference category)			
Equal to average	0.37	0.18–0.77	<0.05
Above average	0.79	0.26–2.39	NS
BMI			
Normal weight (reference category)			
Overweight	0.72	0.17–3.12	NS
Obese	0.40	0.07–2.25	NS
Smoking status			
Not an active smoker (reference category)			
Current smoker	0.32	0.14–0.69	<0.004

## Data Availability

The original contributions presented in this study are included in the article. Further inquiries can be directed to the corresponding author.

## Supplementary Materials

The following supporting information can be downloaded at: https://www.mdpi.com/article/10.3390/nu17111777/s1, Table S1: Proportion of participants adhering to the Mediterranean diet by income level (below average vs. Equal to average).

## Appendix A

**Table A1:** Operational Definitions of Assessment Variables. A culturally sensitive questionnaire in Arabic indicating the following variables.

Name of Variable	Question/Information	Scale	Unit
Independent variables			
Anthropometric measures: Height, weight, and waist circumference BMI	Height measured at baseline Weight measured at baseline Waist circumference (WC)	Continuous Continuous Continuous	Meter Kg cm
	(weight/height^2^)	Composite scale	Kg/m^2^
Dependant variables			
I-MEDAS	Total score calculated based on the responses for each of the MedDiet components	Continuous	
Dietary patterns Olive oil intake	Do you use olive oil as the main source of culinary oil? Do you use olive oil as the main source of added oil (on labaneh, hummus, za’atar)?	Dichotomous	Yes/no
Vegetable intake	How many units of vegetables do you consume per day? (one unit: 1 piece of cucumber, tomato, or sweet pepper.	Continuous	
Fruit intake	How many units of fruit do you consume per day? (one unit: 1 piece of apple, pear, a small branch of grapes, a slice of watermelon)	Continuous	
Fish intake	How often have you been consuming fish per week for the past three months	Continuous	
Soft drinks consumption	Do you usually drink sugary drinks, including fruit juices, cola, tamharindi, and energy drinks at least once a day?	Dichotomous	Yes/no
Dietary drinks	Do you usually drink diet drinks, saccharine, or sugar alcohol sweetened drinks at least once a day?	Dichotomous	Yes/no
Saturated fat consumption	Do you usually eat pita bread, bagels, pastries, waffles, burekas, croissants, or snacks made from margarine at least once a day? If yes, how many meals? (1 meal = ½ pita bread, 1/6 Laffah, 1 small burekas, 1 small croissant)	Dichotomous Continuous	Yes/no
Added sugars intake	Do you usually consume sweets such as kunafa, baqlava, ma’moul, other Arabic sweets, cakes, and cookies at least five days a week	Dichotomous	
Whole grains	Do you usually consume whole grains, such as whole wheat bread or pasta, bulgur, freekah, buckwheat, and groats, per day? If yes, how many meals? If yes, how many units per day? (one unit = 1 slice of bread, ½ pita bread, ½ cup cooked cereals = 3 cooked table spoons)	Dichotomous Continuous	Yes/no
Seeds and nuts intake	Do you usually consume almonds, nuts, or peanuts per week? If yes, how many meals per week? (one meal = 30g or handful)	Dichotomous Continuous	Yes/no
Trans fat consumption	Do you usually consume salty snacks such as chips or roasted seeds? If yes, how many meals per week? (one meal = 1 individual package of salted snacks, or a handful of seeds)	Dichotomous Continuous	Yes/no
Covariables			
Sex	Sex at the examination	Dicohotomous	Male/female
Age	Age at the examination	Continuous	Years
Household perceived Socioeconomic status (SES)	Relatively to those who surround you, what is your average income?	Ordinal	Similar to the average Above average Below average
Education	Number of years spent in education (including high school) What is the highest degree you have obtained	Continuous Ordinal	Years High school graduation certificate. High school diploma Post-secondary school graduation certificate (non-academic). Bachelor’s degree. Master’s degree. Doctoral degree. Other certificate. Please specify Did not receive a certificate.
Occupation	What was your job over the past few years?	Ordinal	Employed as a salaried worker. Self-employed. Student who works. Student who does not work. Working as a family member without pay. Not working due to illness/disability/impairment. Unemployed. Retiree. Housewife.
Smoking	Do you smoke?	Ordinal	Current smokerFormer smokerNever
Baseline physical activity level as measured by a pedometer	Daily steps on a weekly average recorded by a pedometer	Continuous	Steps
Self-reported physical activity	Do you engage yourself in physical activity at least once a week?	Dichotomos	Yes/no
