# Supplementary material for: Adherence to Mediterranean Diet Among Prediabetic Patients in East Jerusalem
Source: Nutrients. 2025 May 23;17(11):1777. doi: 10.3390/nu17111777 (PMC12157735; doi:10.3390/nu17111777)
Supplement: Supplementary file 1 [file nutrients-17-01777-s001.zip › nutrients-3636259-supplementary.pdf]

Supplementary Materials

Table S1. Proportion of participants adhering to the Mediterranean diet by income level (below average vs. Equal to average)

	**Income**			
	**Below Average n (%)**	**Equal to Average n (%)**	**Total Number**	***p*-Value**
Use of olive oil as main source of culinary oil	92 (94.8)	56 (100)	148	0.084
Prefer white meat and poultry over red and processed meat	60 (61.9)	30 (53.6)	90	NS
Vegetables ≥ 2 servings per day	58 (59.8)	29 (51.8)	87	NS
Fruits ≥ 3 servings per day	35 (36.1)	17 (30.4)	52	NS
Butter or margarine< 1 serving each day	8 (8.2)	4 (7.1)	141	NS
Carbonated/ sweetened drinks < 1 serving per day	36 (37.1)	28 (50.0)	89	NS
Whole grains ≥ 3 servings per day	18 (18.6)	10 (17.9)	28	NS
Unsweetened dairy products ≥ 2 servings per day	54 (55.7)	31 (55.4)	85	NS
Red or processed meat< 7 servings per week	2 (2.1)	5 (8.9)	146	0.05
Legumes ≥ 3 servings per week	7 (7.2)	6 (10.7)	13	NS
Fish greater ≥ to 3 servings per week	3 (3.1)	6 (10.7)	9	0.054
Nuts ≥ 3 servings per week	52 (53.6)	56 (50.0)	80	NS
Humus or tahini ≥ to 3 servings per week	40 (41.2)	23 (41.1)	63	NS
Sweet baked goods < 3 servings per week	46 (47.4)	27 (48.2)	80	NS
Savory baked pastries < to 2 per week	22 (22.7)	13 (23.2)	118	NS
Consume salty snacks < less than 3 per week	6 (6.2)	9 (16.1)	138	0.048
Total number	97	56	153	
